# Crystal structure of bis­{(*S*)-1-[2-(di­phenyl­phosphan­yl)ferrocen­yl]-(*R*)-eth­yl}ammonium bromide di­chloro­methane monosolvate

**DOI:** 10.1107/S2056989016020417

**Published:** 2017-01-13

**Authors:** Afrooz Zirakzadeh, Berthold Stöger, Karl Kirchner

**Affiliations:** a Institute of Applied Synthetic Chemistry, TU Wien, Getreidemarkt 9/163, A-1060 Vienna, Austria; b X-Ray Centre, TU Wien, Getreidemarkt 9, A-1060 Vienna, Austria

**Keywords:** crystal structure, ferrocene, PNP ligand, hydrogen bonding

## Abstract

During the synthesis of an FeBr_2_ complex with the PNP ligand (*R*,*R*,*S*_Fc_,*S*_Fc_)-[Fe_2_(C_5_H_5_)_2_(C_38_H_35_NP_2_)] (**1**), single crystals of the di­chloro­methane monosolvate of the Br^−^ salt of the protonated ligand **1**H^+^ were obtained serendipitously, *i.e.* [Fe_2_(C_5_H_5_)_2_(C_38_H_36_NP_2_)]Br·CH_2_Cl_2_. The crystal structure of **1**H·Br·CH_2_Cl_2_ was determined by single-crystal X-ray diffraction. The mean bond lengths in the ferrocene units are Fe—C = 2.049 (3) Å and C—C = 1.422 (4) Å within the cyclo­penta­dienyl rings. The mean C—N bond length is 1.523 (4) Å. The inter­planar angle between the two connected cyclo­penta­dienyl rings is 49.2 (2)°. One ferrocene moiety adopts a staggered conformation, whereas the other is between staggered and eclipsed. The Br^−^ ions and the CH_2_Cl_2_ mol­ecules are located in channels extending along <100>. One ammonium H atom forms a hydrogen bond with the Br^−^ ion [H⋯Br = 2.32 (4) Å and C—H⋯Br = 172 (3)°]. The second ammonium H atom is not involved in hydrogen bonding.

## Abstract 2

The absolute structure of (*R*,*R*,*S*_Fc_,*S*_Fc_)-[Fe_2_(C_5_H_5_)_2_(C_38_H_36_BrNP_2_)]·Br·CH_2_Cl_2_ has been determined by X-ray single-crystal diffraction.

## Chemical context   

**Figure 1:**
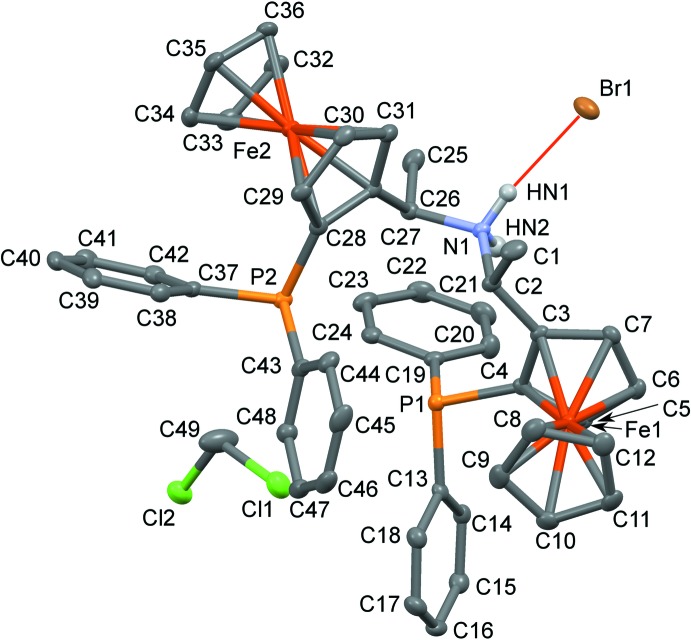
The structures of the molecular entities in **1**H·Br·CH_2_Cl_2_. Non-H atoms are represented by ellipsoids drawn at the 50% probability level (C gray, N blue, P light orange, Cl green, Fe dark orange and Br brown). Ammonium H atoms are represented by white spheres and the hydrogen bond is represented by a red line. Other H atoms have been omitted for clarity.

**Figure 2:**
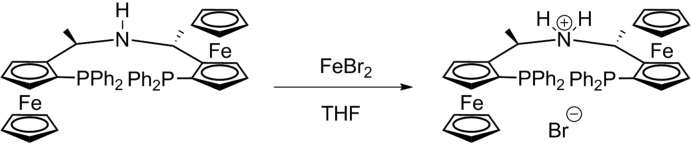
Reaction scheme towards the formation of the title salt **1**H·Br.

During the last decade, chiral non-racemic substituted ferrocene derivatives have found broad applications in a number of different fields, including asymmetric catalysis, and an increasing number of new catalysts and ligands have been reported progressively (Helmchen & Pfaltz, 2000 ▸; Dai *et al.*, 2003 ▸; Sutcliffe & Bryce, 2003 ▸; McManus & Guiry, 2004 ▸; Miyake *et al.*, 2008 ▸; Štěpnička, 2008 ▸; Hargaden & Guiry, 2009 ▸). During the synthesis of chiral PNP pincer ligands [tridentate ligands coordinating to a central metal atom *via* P, N and P (Szabo & Wendt, 2014 ▸)] with a ferrocene scaffold and their Fe^II^ complexes (Hargaden & Guiry, 2009 ▸), the salt **1**H·Br was crystallized as its CH_2_Cl_2_ solvate (Fig. 1 ▸) instead of the expected [Fe(PNP)Br_2_] complex (Fig. 2 ▸). However, neither the crystal structure of any salt of **1**H^+^, nor of any of its solvates, has been reported up to now. The crystal structure of **1**H·Br·CH_2_Cl_2_ is reported in this communication with the aim of contributing to a deeper understanding of its mol­ecular structure and the crystal packing.
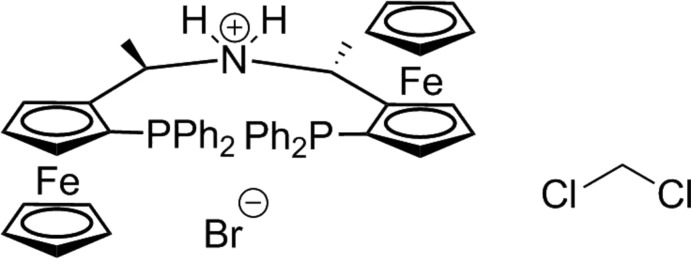


## Structural commentary   

The title salt **1**H·Br crystallizes with one di­chloro­methane mol­ecule in space group *P*4_3_, with one formula unit in the asymmetric unit. The correct space-group assignment, and by consequence absolute configuration, was confirmed by resonant scattering [Flack parameter 0.002 (3); Flack, 1983 ▸]. It is in agreement with the expected absolute configuration as determined by the enanti­oselective synthesis (Zirakzadeh *et al.*, 2016 ▸). In contrast to classical PNP complexes, where the lone pairs of the P and N atoms are directed towards the coordinated metal, the **1**H^+^ ion adopts a distinctly more twisted conformation (Fig. 1 ▸) [the angles of the C—N bonds to the least-squares planes of connected penta­dienyl moieties are 61.2 (2) and 81.9 (10)°]. Whereas the lone pairs of the P atoms are approximately in a face-to-face orientation, the hydrogen atoms of the secondary ammonium group are directed in a different direction towards distinct channels in the structure (see below). The ferrocene moieties adopt staggered (Fe2: average C—G—G—C torsion angle 30.1°, where C stands for a C atom of the ferrocene and G for the center of gravity of the C atoms of the corresponding ring) and somewhat more eclipsed (Fe1: 14.9°) conformations, respectively.

## Supra­molecular features   

**Figure 3:**
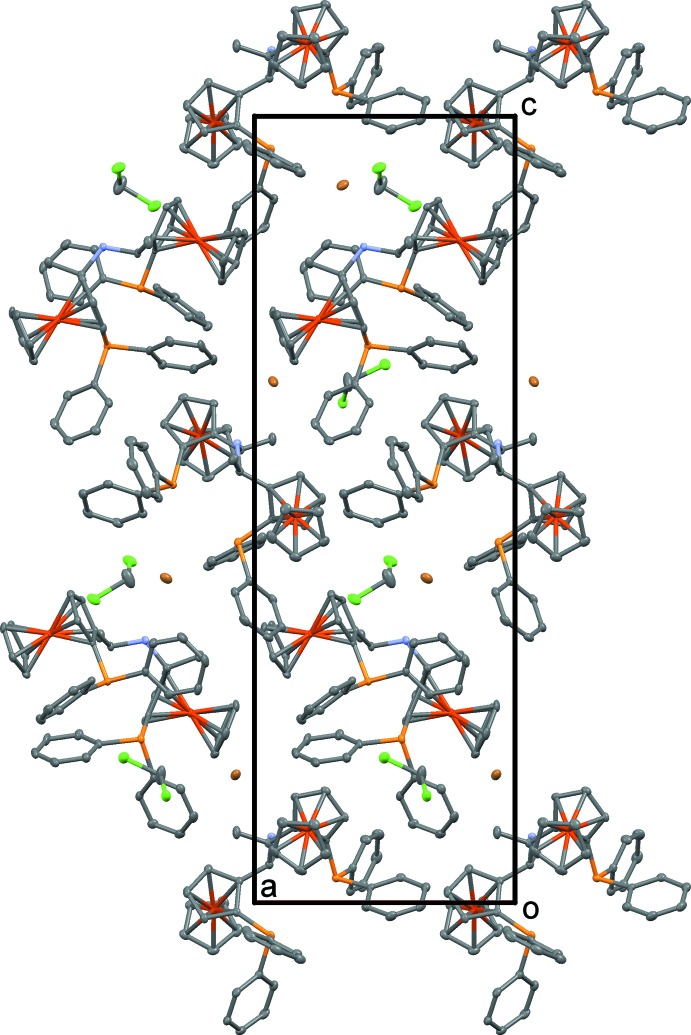
The crystal structure of **1**H·Br·CH_2_Cl_2_ viewed down [010]. Atoms are as in Fig. 1 ▸. H atoms have been omitted for clarity.

One of the two ammonium H atoms forms a hydrogen bond with the Br^−^ ion (Table 1 ▸). The second H atom is not involved in hydrogen bonding. Besides the hydrogen bonding, no further notable supra­molecular inter­actions are apparent. The **1**H^+^ ions form a van der Waals-packed three-dimensional framework (Fig. 3 ▸). The CH_2_Cl_2_ solvent mol­ecules and Br^−^ ions are located in channels of this network that extend along <100>. Without CH_2_Cl_2_ mol­ecules and Br^−^ ions, the packing index (fraction of filled space) is 62.4% [calculated with *PLATON* (Spek, 2009 ▸)]. Each CH_2_Cl_2_ solvent mol­ecule occupies 98 Å^3^ of the structure. In total, the solvent mol­ecules make up a 9.2% volume fraction of the structure.

## Database survey   

A search of the Cambridge Structural Database (Version 5.37; last update March 2016; Groom *et al.*, 2016 ▸) for structures of mol­ecules containing an analogous tridentate ferrocene PNP scaffold revealed no entries. However, three mol­ecules where the secondary amine functionality is replaced by a longer linker were found: AZAHED (amine substituted for imidazolium; Gischig & Togni, 2005 ▸), ALEZMOS (2,6-pyridine dicarboxamide; Reddy *et al.*, 2007 ▸) and PEDTEX (piperazine; Zhou & Zhang, 2005 ▸). Finally, in XARUD (You *et al.*, 2000 ▸) the amine functionality is substituted by a cyclo­hexa­nedi­amine unit. Moreover, the methyl groups are substituted by oxo groups, making XARUD a *bis*-formamide.

## Synthesis and crystallization   

All reactions were performed under an inert atmosphere of argon using Schlenk techniques. The solvents were purified according to standard procedures. The synthesis of **1** and the [Fe(PNP)Br_2_] complex was described in detail by our group (Zirakzadeh *et al.*, 2016 ▸). Single crystals suitable for X-ray structure determination were grown by vapour diffusion of Et_2_O into a CH_2_Cl_2_ solution.

## Refinement   

Crystal data, data collection and structure refinement details are summarized in Table 2 ▸. H atoms bonded to C atoms were placed in calculated positions and refined as riding atoms, with fixed bond lengths in the range 0.95–1.00 Å and *U*_iso_(H) = 1.2*U*_eq_(C) or 1.5*U*_eq_(C_Me_). Ammonium H atoms were found in difference Fourier maps and were refined freely.

## Supplementary Material

Crystal structure: contains datablock(s) I, general. DOI: 10.1107/S2056989016020417/pk2595sup1.cif

Structure factors: contains datablock(s) I. DOI: 10.1107/S2056989016020417/pk2595Isup2.hkl

CCDC reference: 1524191

Additional supporting information:  crystallographic information; 3D view; checkCIF report

## Figures and Tables

**Table 1:** Hydrogen-bond geometry (Å, °)

*D*—H⋯*A*	*D*—H	H⋯*A*	*D*⋯*A*	*D*—H⋯*A*
N1—H*N*1⋯Br1	0.92 (4)	2.32 (4)	3.228 (3)	172 (3)

**Table 2:** Experimental details

Crystal data	
Chemical formula	[Fe_2_(C_5_H_5_)_2_(C_38_H_36_NP_2_)]Br·CH_2_Cl_2_
*M*_r_	975.33
Crystal system, space group	Tetragonal, *P*4_3_
Temperature (K)	100
*a*, *c* (Å)	11.2463 (7), 33.938 (2)
*V* (Å^3^)	4292.5 (6)
*Z*	4
Radiation type	Mo *K*α
μ (mm^−1^)	1.84
Crystal size (mm)	0.35 × 0.17 × 0.11

Data collection	
Diffractometer	Bruker Kappa APEXII CCD
Absorption correction	Multi-scan (*SADABS*; Bruker, 2015 ▸)
*T*_min_, *T*_max_	0.590, 0.746
No. of measured, independent and observed [*I* > 2σ(*I*)] reflections	42331, 12559, 10851
*R*_int_	0.043
(sin θ/λ)_max_ (Å^−1^)	0.704

Refinement	
*R*[*F* ^2^ > 2σ(*F* ^2^)], *wR*(*F* ^2^), *S*	0.032, 0.063, 0.98
No. of reflections	12559
No. of parameters	524
No. of restraints	1
H-atom treatment	H atoms treated by a mixture of independent and constrained refinement
Δρ_max_, Δρ_min_ (e Å^−3^)	0.85, −0.41
Absolute structure	Flack *x* determined using 4530 quotients [(*I* ^+^)−(*I* ^−^)]/[(*I* ^+^)+(*I* ^−^)] (Parsons *et al.*, 2013 ▸)
Absolute structure parameter	0.002 (3)

Computer programs: *APEX2* and *SAINT-Plus* (Bruker, 2015 ▸), *SHELXT* (Sheldrick, 2015*a* ▸), *SHELXL2014* (Sheldrick, 2015*b* ▸), *Mercury* (Macrae *et al.*, 2006 ▸) and *publCIF* (Westrip, 2010 ▸).

## supplementary crystallographic information

### Crystal data

**Table:**

[Fe_2_(C_5_H_5_)_2_(C_38_H_36_NP_2_)]Br·CH_2_Cl_2_	*D*_x_ = 1.509 Mg m^−^^3^
*M**_r_* = 975.33	Mo *K*α radiation, λ = 0.71073 Å
Tetragonal, *P*4_3_	Cell parameters from 9938 reflections
*a* = 11.2463 (7) Å	θ = 2.2–29.6°
*c* = 33.938 (2) Å	µ = 1.84 mm^−^^1^
*V* = 4292.5 (6) Å^3^	*T* = 100 K
*Z* = 4	Tabular, translucent yellow
*F*(000) = 2000	0.35 × 0.17 × 0.11 mm

### Data collection

**Table:**

Bruker Kappa APEXII CCD diffractometer	10851 reflections with *I* > 2σ(*I*)
ω– and φ–scans	*R*_int_ = 0.043
Absorption correction: multi-scan (*SADABS*; Bruker, 2015)	θ_max_ = 30.0°, θ_min_ = 2.2°
*T*_min_ = 0.590, *T*_max_ = 0.746	*h* = −15→15
42331 measured reflections	*k* = −11→15
12559 independent reflections	*l* = −47→47

### Refinement

**Table:**

Refinement on *F*^2^	Hydrogen site location: mixed
Least-squares matrix: full	H atoms treated by a mixture of independent and constrained refinement
*R*[*F*^2^ > 2σ(*F*^2^)] = 0.032	*w* = 1/[σ^2^(*F*_o_^2^) + (0.0078*P*)^2^] where *P* = (*F*_o_^2^ + 2*F*_c_^2^)/3
*wR*(*F*^2^) = 0.063	(Δ/σ)_max_ = 0.003
*S* = 0.98	Δρ_max_ = 0.85 e Å^−^^3^
12559 reflections	Δρ_min_ = −0.41 e Å^−^^3^
524 parameters	Absolute structure: Flack *x* determined using 4530 quotients [(*I*^+^)-(*I*^-^)]/[(*I*^+^)+(*I*^-^)] (Parsons *et al.*, 2013)
1 restraint	Absolute structure parameter: 0.002 (3)

### Special details

**Table:**

Geometry. All esds (except the esd in the dihedral angle between two l.s. planes) are estimated using the full covariance matrix. The cell esds are taken into account individually in the estimation of esds in distances, angles and torsion angles; correlations between esds in cell parameters are only used when they are defined by crystal symmetry. An approximate (isotropic) treatment of cell esds is used for estimating esds involving l.s. planes.

### Fractional atomic coordinates and isotropic or equivalent isotropic displacement parameters (Å^2^)

**Table:**

	*x*	*y*	*z*	*U*_iso_*/*U*_eq_	
C1	−0.0686 (3)	0.4072 (3)	0.58926 (11)	0.0220 (7)	
H1A	−0.1299	0.4497	0.5743	0.033*	
H1B	−0.0781	0.4241	0.6174	0.033*	
H1C	−0.0766	0.3215	0.5847	0.033*	
C2	0.0537 (3)	0.4481 (3)	0.57579 (9)	0.0137 (6)	
H2	0.0604	0.4342	0.5468	0.016*	
C3	0.1551 (3)	0.3877 (3)	0.59565 (9)	0.0132 (6)	
C4	0.2741 (3)	0.3804 (3)	0.57985 (8)	0.0123 (6)	
C5	0.3460 (3)	0.3215 (3)	0.60882 (9)	0.0141 (6)	
H5	0.4322	0.3014	0.6060	0.017*	
C6	0.2736 (3)	0.2951 (3)	0.64204 (9)	0.0164 (7)	
H6	0.3003	0.2527	0.6664	0.020*	
C7	0.1568 (3)	0.3361 (3)	0.63457 (8)	0.0149 (6)	
H7	0.0872	0.3285	0.6527	0.018*	
C8	0.0747 (3)	0.1189 (3)	0.56528 (10)	0.0215 (7)	
H8	−0.0063	0.1489	0.5582	0.026*	
C9	0.1767 (3)	0.1237 (3)	0.54086 (9)	0.0211 (7)	
H9	0.1804	0.1572	0.5136	0.025*	
C10	0.2732 (3)	0.0718 (3)	0.56207 (9)	0.0193 (7)	
H10	0.3566	0.0627	0.5523	0.023*	
C11	0.2304 (3)	0.0365 (3)	0.59963 (9)	0.0195 (7)	
H11	0.2781	−0.0022	0.6209	0.023*	
C12	0.1073 (3)	0.0660 (3)	0.60168 (9)	0.0200 (7)	
H12	0.0533	0.0512	0.6246	0.024*	
C13	0.4461 (3)	0.3477 (3)	0.51945 (8)	0.0142 (6)	
C14	0.5614 (3)	0.3743 (3)	0.53203 (9)	0.0160 (6)	
H14	0.5745	0.4395	0.5493	0.019*	
C15	0.6571 (3)	0.3055 (3)	0.51942 (9)	0.0194 (7)	
H15	0.7355	0.3255	0.5275	0.023*	
C16	0.6386 (3)	0.2085 (3)	0.49530 (9)	0.0215 (7)	
H16	0.7042	0.1612	0.4872	0.026*	
C17	0.5253 (3)	0.1799 (3)	0.48301 (9)	0.0225 (8)	
H17	0.5125	0.1126	0.4666	0.027*	
C18	0.4297 (3)	0.2499 (3)	0.49472 (9)	0.0185 (7)	
H18	0.3520	0.2308	0.4857	0.022*	
C19	0.3763 (3)	0.5819 (3)	0.54381 (9)	0.0149 (6)	
C20	0.4276 (3)	0.6117 (3)	0.57988 (9)	0.0210 (7)	
H20	0.4306	0.5540	0.6003	0.025*	
C21	0.4743 (3)	0.7238 (3)	0.58649 (10)	0.0263 (8)	
H21	0.5103	0.7417	0.6111	0.032*	
C22	0.4687 (3)	0.8100 (3)	0.55753 (11)	0.0247 (8)	
H22	0.5014	0.8867	0.5621	0.030*	
C23	0.4150 (3)	0.7837 (3)	0.52171 (10)	0.0223 (7)	
H23	0.4098	0.8429	0.5018	0.027*	
C24	0.3691 (3)	0.6711 (3)	0.51496 (9)	0.0179 (7)	
H24	0.3322	0.6541	0.4904	0.021*	
C25	0.0812 (3)	0.7888 (3)	0.56353 (10)	0.0212 (7)	
H25A	0.0838	0.8444	0.5413	0.032*	
H25B	0.1578	0.7898	0.5773	0.032*	
H25C	0.0180	0.8125	0.5818	0.032*	
C26	0.0566 (3)	0.6644 (3)	0.54835 (9)	0.0146 (6)	
H26	0.1220	0.6434	0.5296	0.017*	
C27	−0.0585 (3)	0.6444 (3)	0.52736 (9)	0.0130 (6)	
C28	−0.0692 (3)	0.5934 (3)	0.48808 (9)	0.0132 (6)	
C29	−0.1935 (3)	0.5798 (3)	0.48021 (9)	0.0147 (6)	
H29	−0.2285	0.5480	0.4553	0.018*	
C30	−0.2590 (3)	0.6198 (3)	0.51351 (9)	0.0177 (7)	
H30	−0.3476	0.6207	0.5159	0.021*	
C31	−0.1770 (3)	0.6604 (3)	0.54246 (9)	0.0159 (7)	
H31	−0.1981	0.6947	0.5687	0.019*	
C32	−0.1196 (3)	0.9296 (3)	0.50030 (10)	0.0210 (7)	
H32	−0.0771	0.9639	0.5236	0.025*	
C33	−0.0658 (3)	0.8890 (3)	0.46484 (10)	0.0235 (8)	
H33	0.0213	0.8899	0.4588	0.028*	
C34	−0.1575 (3)	0.8482 (3)	0.43933 (9)	0.0211 (7)	
H34	−0.1463	0.8144	0.4123	0.025*	
C35	−0.2676 (3)	0.8630 (3)	0.45922 (9)	0.0199 (7)	
H35	−0.3477	0.8410	0.4487	0.024*	
C36	−0.2440 (3)	0.9129 (3)	0.49689 (9)	0.0200 (7)	
H36	−0.3046	0.9329	0.5174	0.024*	
C37	0.0177 (3)	0.6142 (3)	0.40879 (9)	0.0144 (6)	
C38	−0.0694 (3)	0.5590 (3)	0.38573 (9)	0.0166 (7)	
H38	−0.1047	0.4867	0.3943	0.020*	
C39	−0.1043 (3)	0.6099 (3)	0.35028 (9)	0.0186 (7)	
H39	−0.1645	0.5731	0.3349	0.022*	
C40	−0.0521 (3)	0.7138 (3)	0.33734 (9)	0.0208 (7)	
H40	−0.0783	0.7495	0.3135	0.025*	
C41	0.0376 (3)	0.7662 (3)	0.35875 (10)	0.0222 (7)	
H41	0.0748	0.8364	0.3492	0.027*	
C42	0.0741 (3)	0.7164 (3)	0.39440 (9)	0.0181 (7)	
H42	0.1371	0.7518	0.4089	0.022*	
C43	0.0615 (3)	0.4026 (3)	0.45190 (9)	0.0151 (6)	
C44	−0.0287 (3)	0.3254 (3)	0.46356 (10)	0.0202 (7)	
H44	−0.0985	0.3561	0.4756	0.024*	
C45	−0.0172 (3)	0.2029 (3)	0.45761 (10)	0.0252 (8)	
H45	−0.0790	0.1506	0.4656	0.030*	
C46	0.0841 (4)	0.1580 (3)	0.44007 (10)	0.0246 (8)	
H46	0.0918	0.0748	0.4361	0.030*	
C47	0.1737 (3)	0.2331 (3)	0.42841 (9)	0.0219 (8)	
H47	0.2431	0.2019	0.4162	0.026*	
C48	0.1627 (3)	0.3538 (3)	0.43439 (9)	0.0198 (7)	
H48	0.2254	0.4050	0.4264	0.024*	
C49	0.4840 (4)	0.6399 (4)	0.41211 (16)	0.0437 (11)	
H49A	0.4155	0.6489	0.3940	0.052*	
H49B	0.4773	0.7019	0.4327	0.052*	
HN1	0.021 (3)	0.607 (3)	0.6044 (10)	0.014 (9)*	
HN2	0.136 (4)	0.591 (3)	0.5913 (11)	0.020 (10)*	
N1	0.0665 (3)	0.5811 (2)	0.58352 (7)	0.0139 (5)	
P1	0.31329 (7)	0.43597 (7)	0.53127 (2)	0.01328 (16)	
P2	0.06254 (7)	0.56433 (7)	0.45811 (2)	0.01388 (16)	
Cl1	0.47840 (9)	0.49888 (9)	0.43433 (3)	0.0300 (2)	
Cl2	0.61642 (9)	0.66107 (8)	0.38571 (2)	0.0270 (2)	
Fe1	0.20638 (4)	0.21570 (4)	0.59224 (2)	0.01257 (9)	
Fe2	−0.15647 (4)	0.75393 (4)	0.49136 (2)	0.01342 (10)	
Br1	−0.07213 (3)	0.66115 (3)	0.66249 (2)	0.02449 (9)	

### Atomic displacement parameters (Å^2^)

**Table:**

	*U*^11^	*U*^22^	*U*^33^	*U*^12^	*U*^13^	*U*^23^
C1	0.0133 (17)	0.0182 (18)	0.0344 (18)	−0.0009 (13)	0.0002 (15)	0.0018 (15)
C2	0.0139 (16)	0.0101 (15)	0.0172 (13)	−0.0002 (12)	−0.0008 (12)	−0.0009 (12)
C3	0.0137 (15)	0.0093 (15)	0.0165 (13)	0.0003 (12)	−0.0018 (12)	−0.0006 (12)
C4	0.0129 (16)	0.0090 (15)	0.0151 (12)	−0.0034 (12)	−0.0002 (12)	−0.0026 (11)
C5	0.0134 (16)	0.0106 (15)	0.0184 (14)	−0.0012 (12)	−0.0027 (12)	−0.0011 (12)
C6	0.0209 (17)	0.0133 (16)	0.0151 (13)	−0.0011 (13)	−0.0042 (12)	−0.0005 (12)
C7	0.0169 (17)	0.0142 (16)	0.0136 (12)	−0.0019 (13)	0.0014 (12)	−0.0028 (12)
C8	0.0223 (19)	0.0150 (17)	0.0270 (16)	−0.0029 (14)	−0.0110 (14)	−0.0044 (14)
C9	0.032 (2)	0.0148 (17)	0.0170 (14)	−0.0033 (14)	−0.0065 (14)	−0.0039 (12)
C10	0.0236 (19)	0.0122 (16)	0.0220 (15)	0.0011 (14)	0.0007 (14)	−0.0041 (13)
C11	0.0249 (19)	0.0087 (16)	0.0248 (16)	0.0001 (13)	−0.0044 (14)	0.0026 (13)
C12	0.0239 (19)	0.0123 (16)	0.0236 (15)	−0.0066 (14)	−0.0012 (14)	0.0004 (13)
C13	0.0158 (16)	0.0142 (16)	0.0124 (12)	−0.0009 (13)	0.0040 (12)	0.0016 (12)
C14	0.0202 (17)	0.0134 (16)	0.0146 (13)	−0.0012 (13)	0.0008 (13)	0.0018 (12)
C15	0.0173 (17)	0.0217 (18)	0.0192 (14)	−0.0012 (14)	0.0019 (13)	0.0032 (13)
C16	0.0230 (19)	0.0201 (18)	0.0215 (15)	0.0086 (14)	0.0079 (14)	0.0033 (14)
C17	0.028 (2)	0.0195 (18)	0.0198 (15)	0.0017 (15)	0.0061 (14)	−0.0035 (13)
C18	0.0191 (18)	0.0196 (18)	0.0168 (13)	−0.0024 (14)	0.0008 (13)	−0.0009 (13)
C19	0.0138 (16)	0.0110 (16)	0.0199 (13)	0.0015 (12)	0.0023 (12)	−0.0004 (12)
C20	0.026 (2)	0.0179 (18)	0.0194 (14)	−0.0039 (14)	−0.0016 (14)	0.0025 (13)
C21	0.031 (2)	0.024 (2)	0.0239 (16)	−0.0035 (16)	−0.0003 (15)	−0.0086 (15)
C22	0.026 (2)	0.0141 (17)	0.0345 (18)	−0.0043 (15)	0.0063 (16)	−0.0048 (15)
C23	0.0215 (19)	0.0136 (17)	0.0320 (17)	0.0019 (14)	0.0048 (15)	0.0046 (14)
C24	0.0162 (17)	0.0181 (17)	0.0195 (14)	0.0038 (14)	0.0029 (13)	−0.0002 (13)
C25	0.0213 (19)	0.0170 (18)	0.0251 (16)	−0.0028 (14)	−0.0058 (14)	0.0026 (14)
C26	0.0139 (16)	0.0143 (16)	0.0154 (13)	0.0001 (13)	0.0000 (12)	−0.0003 (12)
C27	0.0124 (15)	0.0117 (15)	0.0150 (13)	0.0000 (12)	−0.0002 (12)	0.0027 (11)
C28	0.0137 (16)	0.0091 (15)	0.0169 (13)	−0.0026 (12)	0.0005 (12)	0.0011 (12)
C29	0.0144 (16)	0.0096 (15)	0.0201 (14)	−0.0015 (12)	−0.0023 (12)	−0.0002 (12)
C30	0.0122 (16)	0.0151 (17)	0.0259 (15)	−0.0023 (13)	0.0003 (13)	0.0049 (13)
C31	0.0151 (17)	0.0140 (16)	0.0187 (14)	0.0014 (13)	0.0019 (12)	0.0035 (12)
C32	0.026 (2)	0.0089 (16)	0.0287 (17)	−0.0016 (14)	−0.0042 (15)	−0.0015 (13)
C33	0.0218 (19)	0.0140 (17)	0.0347 (18)	−0.0017 (14)	0.0061 (15)	0.0057 (15)
C34	0.030 (2)	0.0162 (18)	0.0171 (14)	0.0043 (15)	0.0030 (14)	0.0036 (13)
C35	0.0238 (19)	0.0151 (17)	0.0209 (14)	0.0055 (14)	−0.0052 (14)	0.0015 (13)
C36	0.027 (2)	0.0114 (16)	0.0213 (15)	0.0050 (14)	0.0010 (14)	−0.0005 (13)
C37	0.0144 (16)	0.0114 (15)	0.0173 (13)	0.0019 (12)	0.0027 (12)	0.0013 (12)
C38	0.0167 (17)	0.0135 (16)	0.0196 (14)	−0.0006 (13)	0.0053 (13)	0.0025 (13)
C39	0.0139 (17)	0.0232 (18)	0.0186 (14)	0.0004 (14)	0.0029 (12)	0.0014 (13)
C40	0.0231 (19)	0.0234 (19)	0.0157 (13)	0.0062 (15)	0.0059 (13)	0.0032 (13)
C41	0.0239 (19)	0.0170 (18)	0.0256 (16)	−0.0029 (15)	0.0098 (14)	0.0036 (14)
C42	0.0153 (17)	0.0172 (17)	0.0218 (15)	−0.0009 (14)	0.0025 (13)	−0.0022 (13)
C43	0.0180 (17)	0.0108 (16)	0.0165 (13)	0.0003 (13)	−0.0059 (12)	−0.0016 (12)
C44	0.0187 (18)	0.0175 (18)	0.0245 (15)	0.0019 (14)	−0.0025 (14)	0.0040 (14)
C45	0.032 (2)	0.0130 (17)	0.0310 (17)	−0.0080 (15)	−0.0105 (16)	0.0059 (15)
C46	0.038 (2)	0.0128 (17)	0.0227 (16)	0.0040 (15)	−0.0113 (16)	−0.0026 (14)
C47	0.029 (2)	0.0181 (18)	0.0185 (14)	0.0077 (15)	−0.0044 (14)	−0.0031 (13)
C48	0.0231 (19)	0.0166 (18)	0.0197 (14)	0.0013 (14)	−0.0003 (14)	0.0000 (13)
C49	0.037 (3)	0.021 (2)	0.074 (3)	0.0029 (18)	0.023 (2)	0.008 (2)
N1	0.0150 (15)	0.0117 (13)	0.0150 (12)	0.0027 (11)	−0.0011 (11)	−0.0007 (10)
P1	0.0145 (4)	0.0123 (4)	0.0131 (3)	−0.0007 (3)	−0.0008 (3)	0.0002 (3)
P2	0.0132 (4)	0.0113 (4)	0.0172 (3)	−0.0007 (3)	−0.0011 (3)	−0.0013 (3)
Cl1	0.0394 (6)	0.0249 (5)	0.0257 (4)	−0.0043 (4)	0.0090 (4)	0.0017 (4)
Cl2	0.0363 (6)	0.0189 (4)	0.0258 (4)	−0.0014 (4)	0.0093 (4)	−0.0004 (3)
Fe1	0.0143 (2)	0.0102 (2)	0.01322 (17)	−0.00054 (18)	−0.00217 (17)	0.00136 (17)
Fe2	0.0141 (2)	0.0099 (2)	0.01633 (19)	0.00013 (18)	−0.00018 (18)	0.00051 (17)
Br1	0.02010 (19)	0.0307 (2)	0.02272 (15)	−0.00139 (16)	0.00429 (14)	−0.00784 (15)

### Geometric parameters (Å, º)

**Table:**

C1—C2	1.521 (4)	C25—H25C	0.9800
C1—H1A	0.9800	C26—C27	1.494 (4)
C1—H1B	0.9800	C26—N1	1.521 (4)
C1—H1C	0.9800	C26—H26	1.0000
C2—C3	1.488 (4)	C27—C31	1.439 (4)
C2—N1	1.525 (4)	C27—C28	1.456 (4)
C2—H2	1.0000	C27—Fe2	2.055 (3)
C3—C7	1.443 (4)	C28—C29	1.431 (4)
C3—C4	1.445 (4)	C28—P2	1.826 (3)
C3—Fe1	2.022 (3)	C28—Fe2	2.058 (3)
C4—C5	1.435 (4)	C29—C30	1.422 (4)
C4—P1	1.818 (3)	C29—Fe2	2.038 (3)
C4—Fe1	2.046 (3)	C29—H29	1.0000
C5—C6	1.422 (4)	C30—C31	1.423 (5)
C5—Fe1	2.049 (3)	C30—Fe2	2.042 (3)
C5—H5	1.0000	C30—H30	1.0000
C6—C7	1.415 (5)	C31—Fe2	2.041 (3)
C6—Fe1	2.055 (3)	C31—H31	1.0000
C6—H6	1.0000	C32—C36	1.416 (5)
C7—Fe1	2.051 (3)	C32—C33	1.422 (5)
C7—H7	1.0000	C32—Fe2	2.041 (3)
C8—C9	1.416 (5)	C32—H32	1.0000
C8—C12	1.420 (5)	C33—C34	1.423 (5)
C8—Fe1	2.053 (3)	C33—Fe2	2.039 (3)
C8—H8	1.0000	C33—H33	1.0000
C9—C10	1.427 (5)	C34—C35	1.421 (5)
C9—Fe1	2.055 (3)	C34—Fe2	2.060 (3)
C9—H9	1.0000	C34—H34	1.0000
C10—C11	1.419 (5)	C35—C36	1.421 (4)
C10—Fe1	2.058 (3)	C35—Fe2	2.064 (3)
C10—H10	1.0000	C35—H35	1.0000
C11—C12	1.425 (5)	C36—Fe2	2.050 (3)
C11—Fe1	2.048 (3)	C36—H36	1.0000
C11—H11	1.0000	C37—C38	1.399 (5)
C12—Fe1	2.045 (3)	C37—C42	1.401 (4)
C12—H12	1.0000	C37—P2	1.836 (3)
C13—C18	1.396 (4)	C38—C39	1.389 (4)
C13—C14	1.397 (4)	C38—H38	0.9500
C13—P1	1.837 (3)	C39—C40	1.380 (5)
C14—C15	1.393 (5)	C39—H39	0.9500
C14—H14	0.9500	C40—C41	1.376 (5)
C15—C16	1.380 (5)	C40—H40	0.9500
C15—H15	0.9500	C41—C42	1.394 (5)
C16—C17	1.379 (5)	C41—H41	0.9500
C16—H16	0.9500	C42—H42	0.9500
C17—C18	1.390 (5)	C43—C44	1.393 (5)
C17—H17	0.9500	C43—C48	1.396 (5)
C18—H18	0.9500	C43—P2	1.831 (3)
C19—C20	1.394 (4)	C44—C45	1.398 (5)
C19—C24	1.405 (4)	C44—H44	0.9500
C19—P1	1.838 (3)	C45—C46	1.381 (5)
C20—C21	1.384 (5)	C45—H45	0.9500
C20—H20	0.9500	C46—C47	1.373 (5)
C21—C22	1.382 (5)	C46—H46	0.9500
C21—H21	0.9500	C47—C48	1.378 (5)
C22—C23	1.389 (5)	C47—H47	0.9500
C22—H22	0.9500	C48—H48	0.9500
C23—C24	1.386 (5)	C49—Cl2	1.754 (4)
C23—H23	0.9500	C49—Cl1	1.758 (4)
C24—H24	0.9500	C49—H49A	0.9900
C25—C26	1.516 (4)	C49—H49B	0.9900
C25—H25A	0.9800	N1—HN1	0.92 (4)
C25—H25B	0.9800	N1—HN2	0.83 (4)
			
C2—C1—H1A	109.5	C35—C34—C33	107.8 (3)
C2—C1—H1B	109.5	C35—C34—Fe2	70.00 (18)
H1A—C1—H1B	109.5	C33—C34—Fe2	68.92 (19)
C2—C1—H1C	109.5	C35—C34—H34	126.1
H1A—C1—H1C	109.5	C33—C34—H34	126.1
H1B—C1—H1C	109.5	Fe2—C34—H34	126.1
C3—C2—C1	114.7 (3)	C34—C35—C36	108.1 (3)
C3—C2—N1	107.3 (2)	C34—C35—Fe2	69.70 (19)
C1—C2—N1	109.3 (3)	C36—C35—Fe2	69.25 (18)
C3—C2—H2	108.5	C34—C35—H35	126.0
C1—C2—H2	108.5	C36—C35—H35	126.0
N1—C2—H2	108.5	Fe2—C35—H35	126.0
C7—C3—C4	107.7 (3)	C32—C36—C35	108.1 (3)
C7—C3—C2	127.5 (3)	C32—C36—Fe2	69.42 (19)
C4—C3—C2	124.6 (3)	C35—C36—Fe2	70.32 (18)
C7—C3—Fe1	70.33 (17)	C32—C36—H36	126.0
C4—C3—Fe1	70.09 (17)	C35—C36—H36	126.0
C2—C3—Fe1	129.0 (2)	Fe2—C36—H36	126.0
C5—C4—C3	107.1 (3)	C38—C37—C42	119.1 (3)
C5—C4—P1	130.1 (2)	C38—C37—P2	124.5 (2)
C3—C4—P1	122.8 (2)	C42—C37—P2	116.4 (2)
C5—C4—Fe1	69.62 (18)	C39—C38—C37	119.9 (3)
C3—C4—Fe1	68.32 (17)	C39—C38—H38	120.0
P1—C4—Fe1	126.02 (15)	C37—C38—H38	120.0
C6—C5—C4	108.5 (3)	C40—C39—C38	120.3 (3)
C6—C5—Fe1	69.96 (18)	C40—C39—H39	119.8
C4—C5—Fe1	69.37 (17)	C38—C39—H39	119.8
C6—C5—H5	125.8	C41—C40—C39	120.4 (3)
C4—C5—H5	125.8	C41—C40—H40	119.8
Fe1—C5—H5	125.8	C39—C40—H40	119.8
C7—C6—C5	108.7 (3)	C40—C41—C42	120.2 (3)
C7—C6—Fe1	69.68 (17)	C40—C41—H41	119.9
C5—C6—Fe1	69.50 (17)	C42—C41—H41	119.9
C7—C6—H6	125.6	C41—C42—C37	119.9 (3)
C5—C6—H6	125.6	C41—C42—H42	120.0
Fe1—C6—H6	125.6	C37—C42—H42	120.0
C6—C7—C3	107.9 (3)	C44—C43—C48	118.0 (3)
C6—C7—Fe1	70.01 (18)	C44—C43—P2	126.2 (3)
C3—C7—Fe1	68.18 (16)	C48—C43—P2	115.8 (3)
C6—C7—H7	126.0	C43—C44—C45	120.4 (3)
C3—C7—H7	126.0	C43—C44—H44	119.8
Fe1—C7—H7	126.0	C45—C44—H44	119.8
C9—C8—C12	108.4 (3)	C46—C45—C44	119.9 (3)
C9—C8—Fe1	69.90 (19)	C46—C45—H45	120.0
C12—C8—Fe1	69.41 (19)	C44—C45—H45	120.0
C9—C8—H8	125.8	C47—C46—C45	120.3 (3)
C12—C8—H8	125.8	C47—C46—H46	119.8
Fe1—C8—H8	125.8	C45—C46—H46	119.8
C8—C9—C10	107.8 (3)	C46—C47—C48	119.8 (4)
C8—C9—Fe1	69.76 (19)	C46—C47—H47	120.1
C10—C9—Fe1	69.80 (18)	C48—C47—H47	120.1
C8—C9—H9	126.1	C47—C48—C43	121.6 (3)
C10—C9—H9	126.1	C47—C48—H48	119.2
Fe1—C9—H9	126.1	C43—C48—H48	119.2
C11—C10—C9	108.0 (3)	Cl2—C49—Cl1	111.8 (2)
C11—C10—Fe1	69.42 (19)	Cl2—C49—H49A	109.2
C9—C10—Fe1	69.58 (19)	Cl1—C49—H49A	109.2
C11—C10—H10	126.0	Cl2—C49—H49B	109.2
C9—C10—H10	126.0	Cl1—C49—H49B	109.2
Fe1—C10—H10	126.0	H49A—C49—H49B	107.9
C10—C11—C12	108.0 (3)	C26—N1—C2	117.5 (2)
C10—C11—Fe1	70.14 (19)	C26—N1—HN1	112 (2)
C12—C11—Fe1	69.49 (19)	C2—N1—HN1	113 (2)
C10—C11—H11	126.0	C26—N1—HN2	104 (3)
C12—C11—H11	126.0	C2—N1—HN2	106 (3)
Fe1—C11—H11	126.0	HN1—N1—HN2	103 (3)
C8—C12—C11	107.8 (3)	C4—P1—C13	102.08 (14)
C8—C12—Fe1	70.0 (2)	C4—P1—C19	100.99 (14)
C11—C12—Fe1	69.77 (18)	C13—P1—C19	102.68 (14)
C8—C12—H12	126.1	C28—P2—C43	103.69 (15)
C11—C12—H12	126.1	C28—P2—C37	103.33 (14)
Fe1—C12—H12	126.1	C43—P2—C37	101.34 (14)
C18—C13—C14	118.3 (3)	C3—Fe1—C12	128.57 (14)
C18—C13—P1	116.7 (2)	C3—Fe1—C4	41.59 (12)
C14—C13—P1	124.9 (2)	C12—Fe1—C4	168.69 (13)
C15—C14—C13	120.3 (3)	C3—Fe1—C11	166.34 (13)
C15—C14—H14	119.8	C12—Fe1—C11	40.74 (14)
C13—C14—H14	119.8	C4—Fe1—C11	150.05 (13)
C16—C15—C14	120.4 (3)	C3—Fe1—C5	69.36 (13)
C16—C15—H15	119.8	C12—Fe1—C5	148.54 (13)
C14—C15—H15	119.8	C4—Fe1—C5	41.02 (12)
C17—C16—C15	120.2 (3)	C11—Fe1—C5	115.89 (13)
C17—C16—H16	119.9	C3—Fe1—C7	41.49 (12)
C15—C16—H16	119.9	C12—Fe1—C7	106.61 (13)
C16—C17—C18	119.7 (3)	C4—Fe1—C7	69.37 (12)
C16—C17—H17	120.1	C11—Fe1—C7	126.83 (13)
C18—C17—H17	120.1	C5—Fe1—C7	68.45 (13)
C17—C18—C13	121.1 (3)	C3—Fe1—C8	109.07 (13)
C17—C18—H18	119.4	C12—Fe1—C8	40.54 (13)
C13—C18—H18	119.4	C4—Fe1—C8	131.05 (13)
C20—C19—C24	117.7 (3)	C11—Fe1—C8	68.17 (14)
C20—C19—P1	125.3 (3)	C5—Fe1—C8	169.44 (13)
C24—C19—P1	117.0 (2)	C7—Fe1—C8	117.77 (14)
C21—C20—C19	121.2 (3)	C3—Fe1—C9	118.93 (13)
C21—C20—H20	119.4	C12—Fe1—C9	68.27 (14)
C19—C20—H20	119.4	C4—Fe1—C9	109.98 (13)
C22—C21—C20	120.5 (3)	C11—Fe1—C9	68.29 (13)
C22—C21—H21	119.8	C5—Fe1—C9	130.53 (14)
C20—C21—H21	119.8	C7—Fe1—C9	151.94 (14)
C21—C22—C23	119.5 (3)	C8—Fe1—C9	40.34 (14)
C21—C22—H22	120.2	C3—Fe1—C6	69.05 (12)
C23—C22—H22	120.2	C12—Fe1—C6	115.44 (13)
C24—C23—C22	120.1 (3)	C4—Fe1—C6	68.83 (12)
C24—C23—H23	120.0	C11—Fe1—C6	106.15 (13)
C22—C23—H23	120.0	C5—Fe1—C6	40.54 (12)
C23—C24—C19	121.0 (3)	C7—Fe1—C6	40.32 (13)
C23—C24—H24	119.5	C8—Fe1—C6	149.55 (14)
C19—C24—H24	119.5	C9—Fe1—C6	167.46 (14)
C26—C25—H25A	109.5	C3—Fe1—C10	152.29 (13)
C26—C25—H25B	109.5	C12—Fe1—C10	68.23 (14)
H25A—C25—H25B	109.5	C4—Fe1—C10	118.27 (13)
C26—C25—H25C	109.5	C11—Fe1—C10	40.44 (13)
H25A—C25—H25C	109.5	C5—Fe1—C10	108.27 (14)
H25B—C25—H25C	109.5	C7—Fe1—C10	165.26 (13)
C27—C26—C25	117.3 (3)	C8—Fe1—C10	67.96 (15)
C27—C26—N1	110.1 (3)	C9—Fe1—C10	40.62 (13)
C25—C26—N1	106.8 (2)	C6—Fe1—C10	128.06 (14)
C27—C26—H26	107.4	C29—Fe2—C33	137.41 (13)
C25—C26—H26	107.4	C29—Fe2—C32	177.84 (13)
N1—C26—H26	107.4	C33—Fe2—C32	40.80 (13)
C31—C27—C28	107.4 (3)	C29—Fe2—C31	68.86 (13)
C31—C27—C26	127.8 (3)	C33—Fe2—C31	144.63 (14)
C28—C27—C26	124.6 (3)	C32—Fe2—C31	113.29 (13)
C31—C27—Fe2	68.90 (17)	C29—Fe2—C30	40.80 (13)
C28—C27—Fe2	69.37 (17)	C33—Fe2—C30	174.39 (14)
C26—C27—Fe2	131.1 (2)	C32—Fe2—C30	140.80 (14)
C29—C28—C27	107.1 (3)	C31—Fe2—C30	40.78 (13)
C29—C28—P2	132.0 (2)	C29—Fe2—C36	139.24 (14)
C27—C28—P2	120.9 (2)	C33—Fe2—C36	68.33 (14)
C29—C28—Fe2	68.79 (18)	C32—Fe2—C36	40.51 (14)
C27—C28—Fe2	69.17 (17)	C31—Fe2—C36	108.50 (13)
P2—C28—Fe2	125.03 (16)	C30—Fe2—C36	109.85 (14)
C30—C29—C28	108.9 (3)	C29—Fe2—C27	69.13 (12)
C30—C29—Fe2	69.77 (18)	C33—Fe2—C27	116.16 (14)
C28—C29—Fe2	70.31 (18)	C32—Fe2—C27	112.51 (13)
C30—C29—H29	125.5	C31—Fe2—C27	41.14 (12)
C28—C29—H29	125.5	C30—Fe2—C27	68.97 (13)
Fe2—C29—H29	125.5	C36—Fe2—C27	136.55 (13)
C29—C30—C31	108.3 (3)	C29—Fe2—C28	40.91 (12)
C29—C30—Fe2	69.43 (18)	C33—Fe2—C28	113.02 (14)
C31—C30—Fe2	69.57 (18)	C32—Fe2—C28	139.47 (13)
C29—C30—H30	125.8	C31—Fe2—C28	69.39 (12)
C31—C30—H30	125.8	C30—Fe2—C28	68.98 (13)
Fe2—C30—H30	125.8	C36—Fe2—C28	177.83 (12)
C30—C31—C27	108.3 (3)	C27—Fe2—C28	41.46 (12)
C30—C31—Fe2	69.65 (18)	C29—Fe2—C34	109.54 (13)
C27—C31—Fe2	69.96 (17)	C33—Fe2—C34	40.61 (14)
C30—C31—H31	125.8	C32—Fe2—C34	68.31 (13)
C27—C31—H31	125.8	C31—Fe2—C34	173.18 (14)
Fe2—C31—H31	125.8	C30—Fe2—C34	133.84 (14)
C36—C32—C33	108.0 (3)	C36—Fe2—C34	68.09 (13)
C36—C32—Fe2	70.07 (19)	C27—Fe2—C34	145.19 (13)
C33—C32—Fe2	69.54 (19)	C28—Fe2—C34	114.07 (13)
C36—C32—H32	126.0	C29—Fe2—C35	110.46 (13)
C33—C32—H32	126.0	C33—Fe2—C35	68.08 (15)
Fe2—C32—H32	126.0	C32—Fe2—C35	68.05 (14)
C32—C33—C34	108.0 (3)	C31—Fe2—C35	133.38 (14)
C32—C33—Fe2	69.66 (19)	C30—Fe2—C35	106.95 (14)
C34—C33—Fe2	70.47 (19)	C36—Fe2—C35	40.43 (12)
C32—C33—H33	126.0	C27—Fe2—C35	174.50 (13)
C34—C33—H33	126.0	C28—Fe2—C35	141.44 (13)
Fe2—C33—H33	126.0	C34—Fe2—C35	40.30 (13)

### Hydrogen-bond geometry (Å, º)

**Table:**

*D*—H···*A*	*D*—H	H···*A*	*D*···*A*	*D*—H···*A*
N1—H*N*1···Br1	0.92 (4)	2.32 (4)	3.228 (3)	172 (3)
